# Particulate Organic Matter Dynamics in a Permafrost Headwater Stream and the Kolyma River Mainstem

**DOI:** 10.1029/2019JG005511

**Published:** 2020-02-21

**Authors:** Lisa Bröder, Anya Davydova, Sergey Davydov, Nikita Zimov, Negar Haghipour, Timothy I. Eglinton, Jorien E. Vonk

**Affiliations:** ^1^ Department of Earth Sciences Vrije Universiteit Amsterdam Amsterdam The Netherlands; ^2^ Geological Institute Swiss Federal Institute of Technology (ETH) Zürich Switzerland; ^3^ Northeast Science Station, Pacific Geographical Institute, Far East Branch Russian Academy of Sciences Cherskiy, Republic of Sakha Russia; ^4^ Laboratory of Ion Beam Physics Swiss Federal Institute of Technology (ETH) Zürich Switzerland

**Keywords:** particulate organic carbon, permafrost, Kolyma, carbon isotopes, lipid biomarkers, Arctic

## Abstract

Ongoing rapid arctic warming leads to extensive permafrost thaw, which in turn increases the hydrologic connectivity of the landscape by opening up subsurface flow paths. Suspended particulate organic matter (POM) has proven useful to trace permafrost thaw signals in arctic rivers, which may experience higher organic matter loads in the future due to expansion and increasing intensity of thaw processes such as thermokarst and river bank erosion. Here we focus on the Kolyma River watershed in Northeast Siberia, the world's largest watershed entirely underlain by continuous permafrost. To evaluate and characterize the present‐day fluvial release of POM from permafrost thaw, we collected water samples every 4–7 days during the 4‐month open water season in 2013 and 2015 from the lower Kolyma River mainstem and from a small nearby headwater stream (Y3) draining an area completely underlain by Yedoma permafrost (Pleistocene ice‐ and organic‐rich deposits). Concentrations of particulate organic carbon generally followed the hydrograph with the highest concentrations during the spring flood in late May/early June. For the Kolyma River, concentrations of dissolved organic carbon showed a similar behavior, in contrast to the headwater stream, where dissolved organic carbon values were generally higher and particulate organic carbon concentrations lower than for Kolyma. Carbon isotope analysis (δ^13^C, Δ^14^C) suggested Kolyma‐POM to stem from both contemporary and older permafrost sources, while Y3‐POM was more strongly influenced by in‐stream production and recent vegetation. Lipid biomarker concentrations (high‐molecular‐weight *n*‐alkanoic acids and *n*‐alkanes) did not display clear seasonal patterns, yet implied Y3‐POM to be more degraded than Kolyma‐POM.

## Key Points

- A permafrost‐underlain large arctic river and a first‐orderstream in NE Siberia were sampled every 4–7 days during the open water season
- Contrasting seasonal patterns and concentrations of particulate and dissolved organic carbon are observed for the two catchments
- Kolyma particulate organic matter is generally older, yet according to molecular proxies less degraded thanmaterial from the small stream

## 1 Introduction

Unprecedented warming at high latitudes, presently twice as fast as the global average, has the potential to affect global biogeochemical cycles (Richter‐Menge et al., 2018). The thaw of frozen soils (permafrost) may trigger a positive feedback loop, since permafrost soils store about half of the global soil organic matter (OM) (Hugelius et al., 2014; Schuur et al., 2015), which upon thaw is exposed to decomposition, thereby generating greenhouse gases that fuel further global warming. Permafrost thaw occurs both gradually from the top down and through more abrupt thaw processes (thermokarst and thermoerosion), leading to collapsing landscapes. The extent of thermokarst disturbances is predicted to grow across arctic landscapes, resulting in increased hydrologic conductivity and potentially enhanced release of OM to aquatic environments (e.g., Olefeldt et al., 2016). Hydrological changes, such as higher contributions of groundwater and base flow, predicted as a consequence of permafrost thaw have already been observed (Walvoord et al., 2012; Walvoord & Striegl, 2007), yet their effect on the OM loads of arctic rivers remains unclear. Increasing fluxes of dissolved organic carbon (DOC) and inorganic constituents were measured for the Mackenzie River from 1972–2012 (Tank et al., 2016), whereas a decrease in DOC and bicarbonate discharge with progressing thaw was reported for the Yukon River, possibly caused by enhanced OM decomposition in the soils or sorption to mineral soils (Striegl et al., 2005).

The remobilization of soil OM by abrupt thaw processes such as retrogressive thaw slumps, active layer detachment slides, and thermo‐erosion of river banks and coastlines mostly releases OM in the particulate form (Guo et al., 2007; Guo & Macdonald, 2006; Wild et al., 2019). Suspended particulate OM (POM) is operationally defined as the OM fraction that is collected on a filter (common cut‐off 0.7 μm pore size), while the dissolved OM (DOM) fraction passes through the filter. In arctic rivers, POM is on average older than DOM, and may thus be used as a tracer for OM originating from a permafrost thaw source (e.g., Guo et al., 2007; Wild et al., 2019). Ancient DOM released from ice‐rich Yedoma deposits (Pleistocene ice‐ and OM‐rich loess deposits, Zimov et al., 2006) has been shown to readily degrade upon thaw (Spencer et al., 2015; Vonk et al., 2013). For permafrost‐derived POM, however, its fate remains less clear. During transport via hydraulic pathways, i.e., from headwaters and small streams to larger rivers and the Arctic Ocean, there are two major trajectories for remobilized permafrost POM: (i) continued mineralization and release as CO_2_ and/or CH_4_ to the atmosphere (thereby fueling further warming) or (ii) burial and sequestration in river, lake, or ocean sediments. The fate of POM during lateral transport may thus determine the strength of the permafrost‐carbon feedback to climate, but is currently afflicted with high uncertainties (Vonk & Gustafsson, 2013).

Recent instrumental advances have enabled rapid characterization of DOM using techniques such as absorbance/fluorescence spectroscopy and high‐resolution mass spectrometry (e.g., Mann et al., 2012; Mann et al., 2016; Spencer et al., 2015; Stubbins et al., 2016). Using a combination of UV‐visible absorption measurements, excitation‐emission‐matrices, and DOM incubation experiments, Mann et al. (2012) found that predominantly labile DOM is exported by the Kolyma River during the spring flood. Accordingly, Spencer et al. (2015) observed permafrost‐specific molecular fingerprints by means of Fourier transform ion cyclotron resonance mass spectrometry. In their study, the permafrost thaw stream DOM was both significantly older than the bulk DOM of the Kolyma river and more readily susceptible to biological degradation in incubation experiments. On the other hand, Stubbins et al. (2016) detected relatively low photolability of Yedoma‐derived DOM. While these studies have yielded new insights into the sources and fate of DOM, the composition of arctic river POM has proven more elusive as it cannot be analyzed with the aforementioned tools. Particularly for the Siberian arctic rivers, POM characterization is thus far limited to its bulk carbon isotopic composition (e.g., McClelland et al., 2016; Wild et al., 2019).

For this study, we focus on the POM fraction transported by the Kolyma River mainstem close to its delta near Cherskiy, and from a nearby small headwater catchment (Y3) draining Yedoma permafrost soils. As the active layer thaws over summer, hydrological flow paths change and enable transport of different carbon pools. To evaluate seasonal differences in carbon delivery to the river, we collected water samples every 4–7 days from the ice‐free period (late May to early October) in 2013 and 2015. Concentrations of DOC and particulate organic carbon (POC) along with stable and radiocarbon (δ^13^C, Δ^14^C) isotopes and source‐specific molecular biomarker compositions (leaf‐wax lipids: long‐chain *n*‐alkanoic acids and *n*‐alkanes) of the POC fraction are employed to assess contributions from different sources (contemporary terrestrial versus deeper permafrost or Yedoma), as well as their qualitative degradation state. This high‐temporal‐resolution sampling combined with isotopic fingerprinting and extensive geochemical analysis sheds light on the characteristics and dynamics of present‐day fluvial POM over the thaw season in a major arctic watershed.

## 2 Materials and Methods

### 2.1 Study Area and Sample Collection

The Kolyma River watershed in Northeast Siberia is the world's largest watershed (653,000 km^2^) entirely underlain by continuous permafrost (Holmes et al., 2012). As for all arctic rivers, its discharge is characterized by a strong seasonality. The highest fluxes occur during the spring flood in late May to early June, following river‐ice breakup and snow melt. About 50% of the annual DOM and POM fluxes of the six great arctic rivers (Ob, Yenisey, Lena, Kolyma, Yukon, and Mackenzie) are delivered to the Arctic Ocean during the 2 months of spring (May–June); for the Kolyma, these values are 55% and 59% for DOM and POM, respectively (Holmes et al., 2012; McClelland et al., 2016). With an annual water discharge of 109 ± 7 km^3^, a DOC flux of 818 Gg (10^9^ g) and a POC flux of 123 ± 19 Gg, the Kolyma River ranks fifth among the rivers discharging into the Arctic Ocean with respect to water discharge and OC fluxes (Holmes et al., 2012; McClelland et al., 2016).

**Figure 1:**
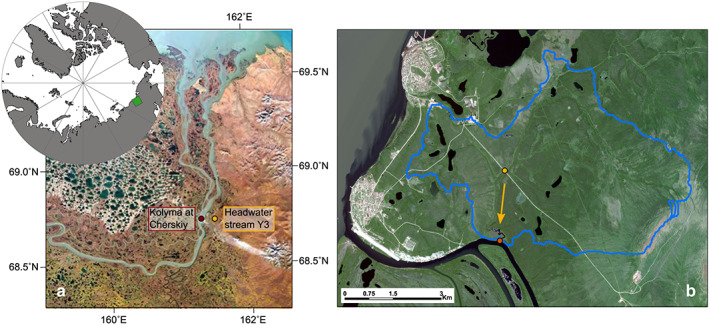
(a) Satellite image of the lower Kolyma river and delta (Sentinel‐2, European Space Agency) with the Kolyma river sampling station close to the town of Cherskiy marked by the red filled circle and the small headwater stream Y3 by the yellow filled circle. The polar projection insert provides an overview with the green box representing the location of the study area. (b) Higher‐resolution satellite image of the Y3 watershed delineated in blue (WorldView‐2 multispectral satellite image from 11 July 2011, courtesy of M. Loranty). Here the yellow‐filled circle marks the sampling site, the yellow arrow the approximate direction of the stream flow, and the orange‐filled circle the outlet of the Y3 stream.

Water samples for this study were collected at two sites in the vicinity of the town of Cherskiy: one representing the Kolyma mainstem (at 68.755°N, 161.305°E) and the other a small headwater stream called Y3 (watershed size ~17 km^2^), draining Yedoma‐rich soils (sampling site at 68.759°N, 161.448°E; Figure 1). Monthly average temperatures at Cherskiy range between −32 °C in January and +13 °C in July with about 200 mm of annual precipitation (2005–2015, for daily average values during the study period see supporting information Figure S1A). Kolyma River breakup usually occurs during the last week of May and the first week of June, and the river starts to freeze up in October.

**Figure 2:**
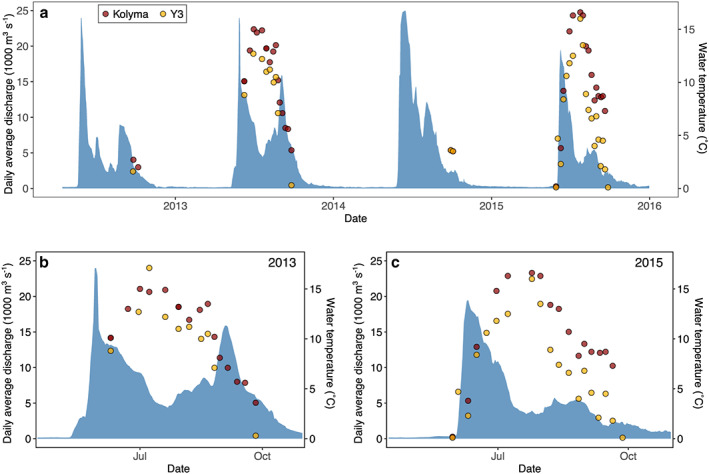
Daily average water discharge of the Kolyma River in blue, water temperatures at the time of sampling for Kolyma in red, Y3 in yellow; (a) for the entire study period, (b) high‐resolution year 2013, (c) high‐resolution year 2015. Discharge data from Roshydromet (Federal Service for Hydrometeorology and Environmental Monitoring, Ministry of Natural Resources and Environment, Russian Federation), measured at Kolymskoye (68.733°N, 158.700°E).

Surface water samples of 5–20 L were collected every 4–7 days from late May until late September/early October to cover the entire open‐water season (Figure 2), resulting in a total of 36 POM samples for the lower Kolyma mainstem and 35 POM samples for headwater stream Y3. Water temperature, pH, and specific conductivity were measured using a YSI Pro‐Plus multiparameter probe. All collected samples were filtered within a few hours after sampling, using precombusted GF/F filters (Whatman, 0.7 μm pore size). The POM fraction collected on the filters was stored frozen, transported to the Netherlands, and then freeze dried. The DOM fraction was acidified to pH 2 with concentrated HCl and stored refrigerated in the dark.

### 2.2 Water Isotopes

Water isotopes (δ^2^H, δ^18^O) were analyzed at ETH‐Zürich (Geological Institute) on a Picarro L2120‐i cavity‐ringdown spectrometer using reference waters from the International Atomic Energy Agency (VSMOW2, GISP, and SLAP2). Filtered samples and reference waters were injected six times. The first two injections were discarded to eliminate instrumental memory effects.

### 2.3 Dissolved OM

All DOC and total dissolved nitrogen concentrations were measured at the Northeast Science Station in Cherskiy through high‐temperature combustion using a Shimadzu TOC‐V organic carbon analyzer linked to a nitrogen chemiluminescence detection unit (TNM‐1). For further method details see Mann et al. (2012).

### 2.4 POM: Elemental and Carbon Isotope Analyses

Concentrations of POC and total particulate nitrogen (TPN) together with stable carbon isotopes (^13^C) were measured at the Stable Isotope Facilities of the University of California at Davis, United States, using an Elementar Vario EL Cube elemental analyzer (EA, Elementar Analysensysteme GmbH, Hanau, Germany) interfaced to a PDZ Europa 20‐20 isotope ratio mass spectrometer (Sercon Ltd., Cheshire, United Kingdom) following their standard procedures. Prior to analysis, freeze‐dried filters were subsampled and acidified repeatedly to remove all inorganic carbon (rinsing with 1 M HCl in precombusted Ag capsules, IVA Analysentechnik GmbH & Co. KG, Meerbusch, Germany). After oven‐drying, they were wrapped in tin capsules (IVA Analysentechnik GmbH & Co. KG, Meerbusch, Germany) to aid the combustion process. All ^13^C data are expressed as delta values (δ^13^C) relative to the international standard VPDB (Vienna PeeDee Belemnite).

Radiocarbon measurements were performed at the Laboratory of Ion Beam Physics of the Swiss Federal Institute of Technology (ETH Zürich, Switzerland). Subsampled filters were fumigated with concentrated HCl (37%, Trace‐Metal purity) at 60 °C for 72 hr to remove carbonates and subsequently dried and neutralized with NaOH at 60 °C for another 72 hr. Measurements were performed on an online EA‐accelerator mass spectrometry (AMS) system (EA: vario MICRO cube, Elementar; AMS: Mini Carbon Dating System MICADAS, Ionplus, Dietikon, Switzerland), for details see Ruff et al. (2010).

### 2.5 POM: Lipid Biomarkers

Freeze‐dried filters containing 1.2 to 17.4 mg of POC (corresponding to 5 to 20 L of filtered water) were solvent‐extracted using a MARS 6 microwave extraction system (CEM, Matthews, North Carolina, United States). Samples were placed in pre‐extracted vessels and extracted twice with 10–15 ml DCM:MeOH (9:1 by volume) at 100 °C (1,600 W, ramp for 5 min, continued heating for 15 min). The resulting total lipid extract was subsequently saponified with 10–15 ml 0.5 M KOH in MeOH (70 °C for 2 hr). After the addition of 5–10 ml MilliQ water with NaCl, the neutral fraction was back‐extracted with hexane (3 × 10 ml). Then the samples were acidified to pH 2 with concentrated HCl and the acid fraction was back‐extracted with hexane: DCM (4:1 by volume). The neutral fraction was further separated into a‐polar and polar fraction by column chromatography (SiO_2_, 5% water‐deactivated, eluting with hexane: DCM, 9:1, and DCM:MeOH, 1:1, respectively). The acid fraction was methylated with BF_3_‐MeOH (80 °C for 30 min) and after the addition of MilliQ water back‐extracted with DCM. The neutral a‐polar fraction (containing *n*‐alkanes) and the acid fraction (containing methylated *n*‐alkanoic acids) were then analyzed on a gas chromatograph fitted with a flame ion detector (Agilent, Santa Clara, California, United States). Quantification of the long‐chain/high‐molecular‐weight *n*‐alkanes and *n*‐alkanoic acids was achieved by comparison with commercial standards (Sigma‐Aldrich/Merck KGaA, Darmstadt, Germany).

## 3 Results

### 3.1 Water Discharge and In Situ Parameters

Water discharge patterns of the Kolyma River displayed distinct differences between the years 2013 and 2015 (Figure 2), which resembles the longer term variability in Kolyma River discharge (McClelland et al., 2016). In 2013, the total discharge was higher than in 2015, with about 115 km^3^ for the period 15 April to 31 October 2013 compared to about 73 km^3^ for the same period in 2015. Besides the prominent peak of the spring flood (or “freshet”) reaching its highest value of 24,000 m^3^ s^−1^ on 29 May 2013, there is a second pronounced peak during late summer, with up to 15,900 m^3^ s^−1^ on 3 and 4 September 2013. In 2015, the freshet peak is smaller and later with a maximum of 19,500 m^3^ s^−1^ on 10 June 2015, and the second peak is also muted (between 4,800 and 5,800 m^3^ s^−1^ from 9 August to 1 September 2015). Water temperatures during the sampling period range between 0.2 and 16.6 °C for Kolyma, and 0.1 and 17.1 °C for Y3 with the highest temperatures recorded in July for both sites and years (Figure 2). Temperatures for Kolyma are on average about 1.4 °C higher than for Y3. For both sites they closely match the daily average air temperature in Cherskiy (Figure S1A).

The pH and specific conductivity data did not display clear seasonal patterns for Kolyma waters but are on average higher than for Y3 (pH: 7.3 and 6.9, specific conductivity: 76 and 55 μS cm^−1^, respectively, Figures S1B and S1C). For both sites pH and specific conductivity are lowest during the freshet. For Y3, the specific conductivity increases from late May to mid‐August, then sharply decreases, followed afterward by a moderate rise again. This pattern is correlated to pH (*R* ^2^ = 0.55, *p* < 0.001).

### 3.2 Water Isotopes (^2^H and ^18^O)

**Figure 3:**
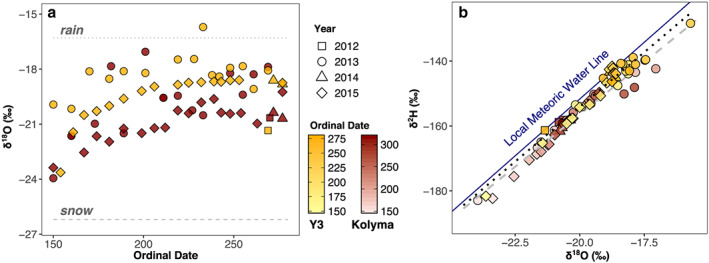
Water isotope values for Kolyma and Y3 samples (filled symbols in red and yellow, respectively) with symbol shapes specifying year of sample collection. (a) Oxygen isotope values (δ^18^O) for both Kolyma and Y3 become more enriched over the open‐water season. In spring, discharge is more influenced by snow, while over the summer rain becomes more dominant. Snow and rain endmember values are resembled by dotted and dashed gray lines, respectively, data from Welp et al. (2005). (b) Relationship between hydrogen (^2^H) and oxygen (^18^O) isotopes, color gradients resembling day of year. The blue line shows the Local Meteoric Water Line (as determined by Welp et al., 2005), the gray dashed line and the black dotted line are the linear fit to the Kolyma and Y3 water samples, respectively (*R* ^2^ = 0.94 and *p* < 0.001 for both).

Water isotope values for Kolyma and Y3 range from −24.0‰ to −17.1‰ for δ^18^O and from −183‰ to −139‰ for δ^2^H (with one exceptionally enriched sample for Y3 on 21 August 2013, where δ^18^O = −15.7‰ and δ^2^H = −128‰). Both locations display a trend from more depleted δ^18^O and δ^2^H values early in the season, i.e., at the beginning of the freshet in late May/early June, to more enriched values in early August. From early August to end of September the isotopic composition remains relatively stable (Figure 3a for ^18^O and Figure S2A for ^2^H isotopes). Values for δ^18^O and δ^2^H are highly correlated for both of the sites (*R* ^2^ = 0.95, *p* < 0.001), with Y3 following the linear relationship δ^2^H = 6.7 * δ^18^O − 19.4‰, and Kolyma δ^2^H = 6.4 * δ^18^O − 27.6‰ (Figure 3b).

### 3.3 DOC and POC and Nitrogen

**Figure 4:**
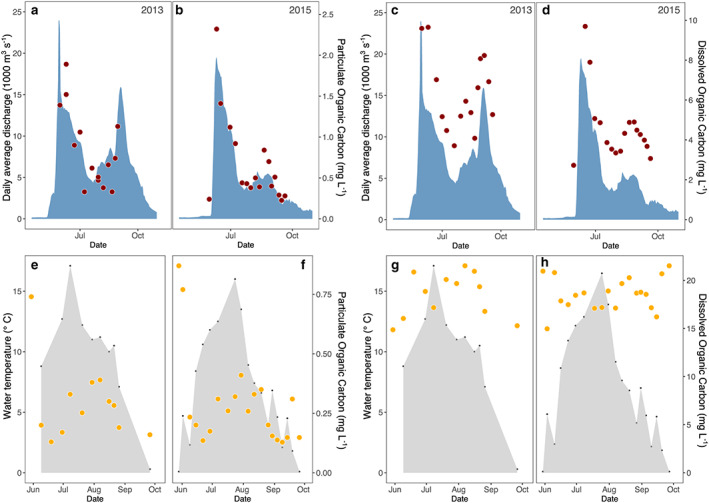
Concentrations of particulate (a, b, e, f) and dissolved (c, d, g, h) organic carbon for Kolyma in red (a–d) and Y3 in yellow (e–h) for the high‐resolution sampling years 2013 (a, c, e, g) and 2015 (b, d, f, h). Daily average water discharge is plotted in blue for the Kolyma and water temperatures in black/gray for Y3 to provide an indication of where the samples are located on the hydrograph/in the season.

Organic carbon concentrations are generally highest during the spring flood (Figure 4). For the Kolyma River (for which discharge data are available), both POC and DOC concentrations correlate with daily average water discharge (*R* ^2^ = 0.67 and *R* ^2^ = 0.87, respectively, *p* < 0.001 for both; Figure S3). POC concentrations range from 0.23 to 2.6 mg L^−1^ (Figures 4a and 4b), while DOC concentrations range from 1.6 to 10.6 mg L^−1^ (Figures 4c and 4d).

**Figure 5:**
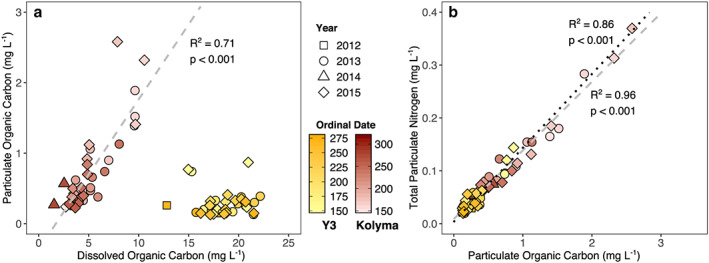
(a) Concentrations of particulate and dissolved organic carbon for the Kolyma River (red) and Y3 headwater stream (yellow). Symbols denote different years and the color scale different days of the year. The gray dashed line shows the linear correlation between particulate organic carbon and dissolved organic carbon for the Kolyma River samples (*R* ^2^ = 0.71, *p* < 0.001). (b) Concentrations of total particulate nitrogen and organic carbon are strongly correlated (Kolyma: gray dashed line, *R* ^2^ = 0.96; Y3: black dotted line, *R* ^2^ = 0.86, *p* < 0.001 for both).

While discharge data are not available for headwater stream Y3, the POC pattern over time seems similar to that of the Kolyma: the highest concentrations are observed at the beginning of the flow season when water temperatures are still close to 0 °C, then concentrations decrease for most of July with a second peak in August (Figures 4e and 4f). POC concentrations for Y3 range from 0.13 to 0.41 mg L^−1^, with DOC concentrations varying between 12.8 and 22.2 mg L^−1^. DOC concentrations for Y3 display no clear seasonal trend (Figures 4g and 4h). Correspondingly, while Kolyma POC and DOC concentrations are correlated with one another (*R* ^2^ = 0.71, *p* < 0.001, Figure 5a) as both correlate with discharge, this relationship does not hold for Y3, where DOC values are generally higher and POC concentrations lower than for Kolyma.

TPN concentrations closely follow the POC concentrations (Figure 5b) and range from 0.02 to 0.14 mg L^−1^ for Y3 and from 0.04 to 0.37 mg L^−1^ for Kolyma. Total dissolved nitrogen concentrations are about one order of magnitude higher than their particulate counterpart (0.35–1.7 mg L^−1^ for Y3 and 0.12–3.3 mg L^−1^ for Kolyma; Figure S4A) and do not correlate with DOC concentrations. This is probably caused by elevated dissolved inorganic nitrogen species (e.g., NO_3_, NO_2_, NH_4_). Inorganic N contributions to the TPN concentration are small, however, as can be deduced from the low intercept of the TPN‐POC correlations (9.0 μg L^−1^ for Kolyma and 3.5 μg L^−1^ for Y3). However, TPN values may also be slightly lowered due to the fact that the measurements were conducted on acidified samples (see section 2.4 for details). Molar ratios of organic carbon and nitrogen (C:N) vary between 5.4 and 10.5 for the Kolyma, and 4.2 and 12.3 for Y3 (Figure S4B).

### 3.4 POC: Isotopic Composition and Molecular Markers

**Figure 6:**
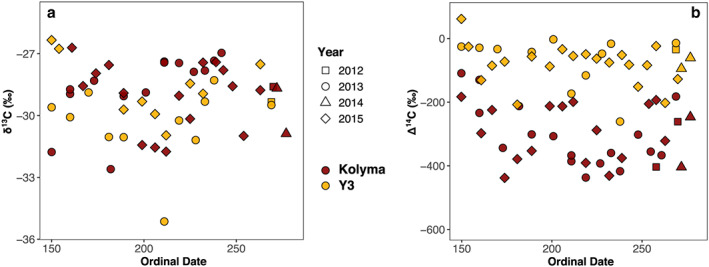
Carbon isotope signatures for particulate organic carbon from Kolyma (red) and Y3 (yellow). (a) Stable carbon isotope values (δ^13^C) do not show clear seasonal trends or differences between sites. (b) Radiocarbon data (Δ^14^C) display a distinct contrast between younger POM for Y3 and older POM for Kolyma.

For Kolyma, POC‐δ^13^C values range from −32.6‰ to −26.7‰. POC‐δ^13^C values for Y3 are similar, falling between −31.2‰ and −26.3‰, with the exception of one very low value (−35.1‰) on 30 July 2013 (Figure 6a). Generally, the POC‐δ^13^C values for Y3 are negatively correlated with water temperature (i.e., higher POC‐δ^13^C values for lower water temperatures; *R* ^2^ = 0.36, *p* < 0.009), pH (*R* ^2^ = 0.46, *p* < 0.003), and specific conductivity (*R* ^2^ = 0.36, *p* < 0.009). Omitting the very low value of −35.1‰ strengthens these correlations. For Kolyma, only a weak negative correlation between δ^13^C and pH was found (i.e., higher POC‐δ^13^C values for lower pH; *R* ^2^ = 0.23, *p* < 0.01).

Δ^14^C values for Kolyma‐POM fall between −438‰ and −109‰, while those for Y3‐POM range from −261‰ to +62‰ (Figure 6b), translating to an average radiocarbon age of ~2,840 and ~590 years, respectively. No seasonal trends in POC‐Δ^14^C are discernable in our data set, yet for both sites the highest Δ^14^C values (i.e., the youngest samples) were measured very early in the season, just before or during the freshet. For Kolyma, Δ^14^C values are negatively correlated with δ^13^C values (i.e., more enriched δ^13^C for older POM, *R* ^2^ = 0.56, *p* < 0.001), which is not the case for Y3.

High‐molecular weight (HMW, i.e., carbon chain lengths of ≥23) *n*‐alkane concentrations range between 0.39 and 2.25 mg gOC^−1^ for Kolyma‐POM (POC‐discharge weighted average of 0.86 mg gOC^−1^) and between 0.16 and 1.71 mg gOC^−1^ for Y3 (POC‐concentration weighted average of 0.46 mg gOC^−1^). For both sites, concentrations appear to be elevated during the spring flood and then again in late summer (Figure S5A), yet no correlation with discharge and only a weak correlation with water temperature (*R* ^2^ = 0.24, *p* < 0.05) was found for Kolyma. The latter did not hold for Y3.

HMW (i.e., carbon chain lengths of ≥24) *n*‐alkanoic acid concentrations for Kolyma‐POM range from 0.16 to 1.24 mg gOC^−1^ with a POC‐discharge weighted average of 0.86 mg gOC^−1^ and display no clear seasonal pattern (Figure S5B). Concentrations for Y3‐POM are generally lower (0.01 to 0.80 mg gOC^−1^, with one high value of 1.36 mg gOC^−1^ on 16 August 2013, and a POC‐concentration weighted average of 0.23 mg gOC^−1^).

## 4 Discussion

### 4.1 Water Sources

Overall, the small headwater stream Y3 exhibited clearer patterns for in situ parameters compared to the Kolyma river, which is likely caused by the fact that for the latter various processes are integrated over the much larger watershed. The observed trends in specific conductivity and pH for Y3 may be caused by a combination of first snow melt (with a low specific conductivity and pH) and then active layer thaw in the early summer (increasing specific conductivity and pH). The later rise from late August to early October may result from the refreezing of the active layer (and the stream eventually), when the remaining liquid waters are enriched in salts/ions. The cause of the sudden drop in specific conductivity each year in early/mid‐August is less clear. In 2013, it coincides with the highest precipitation event; however, this is not the case for 2014 and 2015. Possibly, the steep decrease in specific conductivity reflects the end of the growing season, leading to a reduction in water transpiration of vegetation and thereby increased runoff (decreasing specific conductivity). However, this hypothesis cannot be validated due to the lack of discharge data for Y3.

Water isotopes help to trace contributions of different water sources through the hydrological cycle. Generally, precipitation is more depleted in the heavy stable isotopes (^2^H and ^18^O) at higher latitudes and altitudes and farther inland. Snow is typically more depleted than rain, which was confirmed for precipitation collected in the Cherskiy area by Welp et al. (2005). That study determined isotopic endmember values for δ^18^O of snow and rain to be −26.2 ± 5‰ and −16.3 ± 3.8‰, respectively (Figure 3a). All δ^18^O values of the samples analyzed here fall between these two endmembers. As observed by the increasing pH and specific conductivity, a decreasing contribution from melting snow as a water source is also denoted by the trends toward more enriched water isotopes from spring to summer. Kolyma has generally more depleted isotopic values than Y3, which is probably due to a continental effect: The small Y3 watershed is located relatively close to the Arctic Ocean and thus receives a more marine‐influenced precipitation, whereas the vast Kolyma watershed extends much farther south and thus receives more inland precipitation (and partly also from higher altitudes). For both sites, the linear relationships between δ^2^H and δ^18^O display slightly lower values for the slope (6.7 for Y3 and 6.4 for Kolyma) than for the Local Meteoric Water Line: δ^2^H = 7 * δ^18^O − 11.7‰ (Welp et al., 2005), which may be caused by moderate evaporation in the catchments. Deuterium excess values (d, calculated as d = δ^2^H – 8 * δ^18^O) provide information of humidity conditions in the water source region. Here they show no clear seasonal pattern and are relatively low at both sites (4.2‰ for Kolyma and 4.7‰ for Y3 on average, Figure S2B), corresponding to comparatively high humidity during water vapor formation.

### 4.2 OM Concentrations and Source Proxies

From this data set we determined the discharge‐weighted average of POC for Kolyma to be 1.1 mg L^−1^ (1.0 and 1.3 mg L^−1^ for the separate years 2013 and 2015, respectively), which compares well with an earlier estimate by McClelland et al. (2016) of 1.0 mg L^−1^, where depth‐integrated water samples collected between 2003–2006 and 2009–2012 were analyzed. The average concentration (not discharge‐weighed) is with 0.76 mg L^−1^ higher than the 0.62 mg L^−1^ found in the earlier study. This difference is attributed to a lack of samples for the low concentration winter months in the current study. On the other hand, the time‐resolution of the study by McClelland et al. is considerably lower than for this current investigation (on average five samples per year, versus 19 samples for each open water season 2013 and 2015), and did not always cover the freshet where POC concentrations are particularly high. Nevertheless, a similar correlation of POC concentrations with discharge was observed (*R* ^2^ value of 0.63, McClelland et al., 2016; here *R* ^2^ = 0.71). Our discharge‐weighted average TPN concentration for Kolyma is with 0.15 mg L^−1^ also similar to the earlier estimate of 0.16 mg L^−1^ by McClelland et al. (2016).

The observed differences in POC and DOC concentrations between Y3 and Kolyma may be caused by at least two processes: POC concentrations are likely higher for the Kolyma because this larger river has enough force to erode particles and keep them in suspension, as opposed to the small Y3 stream. The latter is instead dominated by leached DOM, due to its close coupling with underlying soils. For Kolyma, on the other hand, leached DOM may experience substantially longer transport times, allowing for DOM degradation and thus lower overall DOC concentrations (as also demonstrated by Frey et al., 2016).

Molar carbon to nitrogen ratios help to elucidate OM sources, with higher C:N (>10) ratios attributed to terrestrial OM (e.g., Meyers, 1994). The values observed here (5.4 to 10.5 for the Kolyma, 4.2 to 12.3 for Y3) are indicative of OM from aquatic production with some contribution of C3 plant material. The discharge‐weighted average C:N value of 8.4 for Kolyma compares well with that of 8.1 found by McClelland et al. (2016). No clear seasonal patterns are observed for Kolyma or Y3, yet for Kolyma, there is a weak positive correlation with discharge (*R* ^2^ = 0.28, *p* < 0.001), suggesting a higher contribution of C3 plant material with elevated discharge.

Carbon isotopes also carry information of the OM source and have been widely used to assess their relative contributions (for the East Siberian Arctic Shelf, e.g., Vonk et al., 2012; for Siberian large rivers, e.g., Wild et al., 2019). Higher (i.e., more ^13^C‐enriched) stable carbon isotope values (δ^13^C) for riverine OM generally indicate a larger proportion of (fresh) terrigenous material, while lower (i.e., more ^13^C‐depleted) values are typically attributed to in‐stream primary production, fueled by CO_2_ from the decomposition of terrigenous OM (e.g., Finlay, 2001; Meyers, 1994). However, since δ^13^C values for POM from aquatic production can vary substantially and may also be influenced by degradative processes during riverine transport (e.g., Wild et al., 2019), they need to be interpreted cautiously.

**Figure 7:**
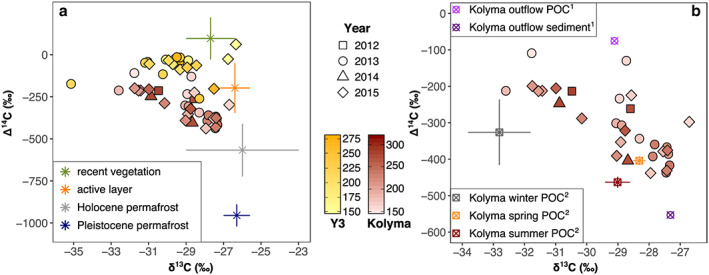
(a) Carbon isotopic fingerprints of the two sites (Kolyma‐POM in red and Y3‐POM in yellow) compared to endmember values for potential OM sources in the study area compiled by Wild et al. (2019). (b) Comparison of Kolyma‐POM with (1) POM and sediments collected just outside the Kolyma delta (Vonk et al., 2010) and (2) seasonal averages for the Kolyma from McClelland et al. (2016). All error bars resemble ±σ.

The observed correlations for δ^13^C with water temperature, pH, and specific conductivity for Y3 may result from the comparatively higher aquatic production of POM in the small stream. Also, the very depleted outlier of −35.1‰ on 30 July 2013 may have been caused by a localized algae bloom, although this is not reflected in any of the other parameters. The relatively high turbidity of the Kolyma river waters (with on average ~35 mg L^−1^ total suspended solids for the summer months, Holmes et al., 2018) likely constrains primary production to the top few centimeters, and the relatively high input of terrigenous material curtails the contribution of aquatic vegetation further. The shift from lower to higher δ^13^C values with increasing discharge that was observed by McClelland et al. (2016) is not visible in this data set. This may be due to the fact that the study period here does not include the winter months (November to April), during which the river is ice‐covered and characterized by very low water flow, and when the lowest δ^13^C values were measured (−32.8 ± 1.0‰, McClelland et al., 2016). For spring and summer, the values measured here agree well with that earlier study: −28.3 ± 0.3‰ for May–June, here −28.7 ± 1.5‰; −29.0 ± 0.4‰ for July–October, here −29.0 ± 1.6‰ (see also Figure 7b).

Radiocarbon analysis of POC provides further insights into OM sources and their relative contributions. Ice‐complex deposit permafrost soils of Northeast Siberia store large amounts of OM from the Pleistocene (e.g., Zimov et al., 2006), resulting in an endmember ^14^C value that is almost radiocarbon‐dead (Δ^14^C = −940 ± 84‰, Vonk et al., 2012). Recent terrestrial and aquatic vegetation, on the other hand, incorporates mostly modern carbon from the atmosphere, potentially even carrying elevated levels of ^14^C affected by nuclear weapons testing during the 1960s and 1970s (Δ^14^C = +97 ± 125‰, Wild et al., 2019).

The overall older ^14^C ages of Kolyma mainstem POM (compared to Y3) may be explained by several factors, including (i) remobilization of deeper soil material within the upper Kolyma where active layer depths are greater, (ii) thermokarst features throughout the watershed that expose deeper, older deposits, and/or (iii) river bank erosion, all delivering old POM from deeper permafrost soils to the river. The small headwater stream Y3, in contrast, does not seem to receive significant amounts of Pleistocene POM, likely because it either does not have the strength to erode deeper soils or its active layer is so shallow that the Yedoma deposits are not exposed. The Y3‐POM is therefore dominated by material from recent vegetation and active layer sources. Despite elevated Δ^14^C values at the beginning of the season, when soils are still mostly frozen solid and river bank erosion sites are largely inactive, we observed no clear seasonal patterns or correlations with, e.g., water temperature for either Kolyma or Y3. A shift toward lower Δ^14^C values over the summer would be expected, as inputs from old, deep soils are thought to be highest at times of maximal thaw in late August/early September, as seen, e.g., in Neff et al. (2006) for DOC. On the other hand, this trend toward lower Δ^14^C values could be counterbalanced by an increase in primary production fixing modern CO_2_ from the atmosphere.

With an average of −260 ± 113‰ and −314 ± 83‰ for spring and summer Kolyma‐POM, respectively, the samples collected for this study were younger and showed a larger variation (n_spring_ = 9, n_summer_ = 29) than average Δ^14^C values reported by McClelland et al. (2016), yet they followed the same general pattern of higher values in spring with −404 ± 12‰, −463 ± 15‰, and −326 ± 90‰ for Kolyma‐POM collected in spring, summer, and winter, respectively (n_spring_ = 13, n_summer_ = 13, n_winter_ = 6). In fact, the lowest Δ^14^C (oldest) value measured here (−438‰) is still higher (younger) than the summer average measured by McClelland et al. (2016). While mainly covering two years (2013 and 2015), compared to seven years in that earlier study (2003–2006 and 2009–2011), we here collected samples with a significantly higher time‐resolution, especially for the summer season. The reason(s) why the POM analyzed here is younger on average remains unclear. One possible explanation is differences in sampling methodology (i.e., surface versus depth‐integrated sampling), with older bottom‐water POM excluded by our surface water sampling approach. However, discharge‐weighed POC concentrations, δ^13^C values, and C:N ratios are very similar for both studies, suggesting no significantly different POM pools in surface and bottom waters.

**Figure 8:**
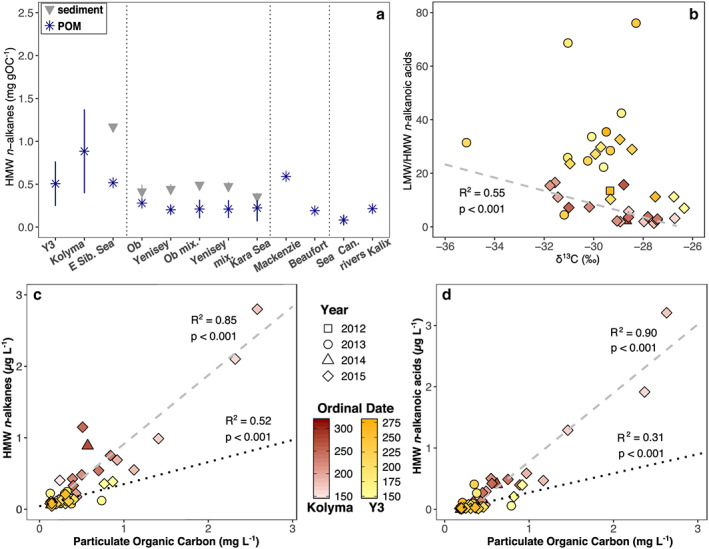
(a) Comparison of average HMW *n*‐alkane concentrations for Y3 and Kolyma from this study to samples from the Kolyma paleoriver (East Siberian Sea, Vonk et al., 2010), Ob and Yenisey river to Kara Sea (Fernandes & Sicre, 2000), Mackenzie and smaller Canadian rivers (Yunker et al., 2002), outside the Mackenzie river delta (Beaufort Sea, Tolosa et al., 2013), and the Kalix river in Northern Sweden (van Dongen et al., 2008). Here blue stars depict POM and gray triangles surface sediment samples; vertical lines resemble ±σ. (b) The ratio of low‐ to high‐molecular‐weight (LMW/HMW) *n*‐alkanoic acids correlates with δ^13^C for Kolyma‐POM (red symbols, gray dashed line), yet not for Y3‐POM (yellow symbols, *p* > 0.45). Per‐liter concentrations of (c) HMW n‐alkanes and (d) HMW n‐alkanoic acids are linearly correlated with POC concentrations with a stronger correlation for Kolyma‐POM (red symbols and gray dashed line, *R* ^2^ = 0.85 and 0.90, respectively, *p* < 0.001) than Y3‐POM (yellow symbols and black dotted line, *R* ^2^ = 0.52 and 0.31, respectively, *p* < 0.001).

Dual‐carbon isotope signatures of potential POM sources (i.e., endmembers), comprising recent vegetation, active layer soil OM, Holocene, and Pleistocene permafrost soil OM, have been compiled in a recent study (Wild et al., 2019; Figure 6c). Their Δ^14^C values range from +97 ± 125‰ for recently formed vegetation to −955 ± 66‰ Pleistocene permafrost deposits along river banks and coastlines and thus cover the entire range of the samples from Kolyma and Y3. Corresponding δ^13^C values, however, span only from −27.7 ± 1.3‰ for recent vegetation to −26 ± 3‰ for Holocene permafrost deposits, while the majority of the Kolyma‐ and Y3‐POM samples are more depleted than these endmembers. This may reflect aquatic production, which is fueled by terrigenous OM and results in lower δ^13^C values through additional fractionation effects (Finlay, 2001; Wild et al., 2019). This assumption appears to be supported by biomarker data (see Figure 8b and discussion there). Based on Δ^14^C data alone, Y3‐POM seems to derive mainly from recent vegetation and active layer soil OM, with minor contributions from Holocene and possibly even Pleistocene permafrost soils, while Kolyma‐POC likely receives greater proportions of older material from deeper parts of the permafrost (as also discussed in the previous paragraph). Petrogenic carbon is another potential endmember for North American arctic rivers (Hilton et al., 2015; Vonk et al., 2016), yet our radiocarbon data and the TPN to POC ratios showed negligible influence of petrogenic POM for these samples.

A comparison of the carbon isotope data gathered here with surface water POM and surface sediment samples collected in the East Siberian Sea, close to the Kolyma delta (station YS‐34B at 46 km distance to the river mouth; 69.71°N, 162.69°E; sampled in early September 2008; Vonk et al., 2010) shows marked differences (see Figure 7b). Coastal surface water POM was significantly younger (Δ^14^C, −75‰) than Kolyma‐POM (−304 ± 94‰) and more similar to Y3‐POM (−100 ± 66‰), while sedimentary OM was older (−553‰) than any of the river samples analyzed here. As suggested in that study, this discrepancy may be caused by preferential burial of aged POM that is potentially ballasted and protected from degradation by its close interaction with the mineral matrix (see also Vonk et al., 2014). Additionally, sediments outside the Kolyma delta likely receive significant amounts of pre‐aged OM supplied directly from the eroding coastline, which is dominated by Pleistocene ice‐complex deposits (Vonk et al., 2010, 2012). The δ^13^C values of −29.1‰ for surface water POM and −27.3‰ for surface sediments outside the Kolyma delta fall within the range covered by Kolyma‐POM collected for this study and are comparable to the POC‐discharge weighted average of −28.6‰.

Lipid biomarkers have been used extensively to trace OM (for the Siberian Arctic, e.g., Bauch et al., 2001; Bröder et al., 2016; Vonk et al., 2010; Zech et al., 2011). Long‐chain or HMW *n*‐alkanes and *n*‐alkanoic acids were found to be useful markers of terrigenous vegetation, as they are almost exclusively produced as leaf waxes of higher plants (Eglinton & Hamilton, 1967). Yet since, to the best of our knowledge, these are the first lipid biomarker data available for POM of our study region, we cannot compare our results to earlier findings for this area. Lipid biomarker data are available for the East Siberian Sea, however, with surface water POM and surface sediments collected within the paleoriver channel close to the Kolyma delta (Vonk et al., 2010; station YS‐34B, see above). These samples displayed HMW *n*‐alkane concentrations of 0.52 mg gOC^−1^ for POM and 1.2 mg gOC^−1^ for surface sediments (Figure 8a). The respective HMW *n*‐alkanoic acid concentrations were 0.57 and 3.7 mg gOC^−1^. Values for POM from that earlier study thus agree well with these new results (Kolyma: 0.88 ± 0.49 mg gOC^−1^, Y3: 0.51 ± 0.26 mg gOC^−1^), whereas the OC‐normalized HMW *n*‐alkanoic acid concentrations in the shelf sediment are higher than any of the values measured here. Corresponding HMW *n*‐alkane concentrations in the sediment are comparable to the highest values measured here.

To allow for a wider comparison with other arctic rivers we have included OC‐normalized HMW *n*‐alkane concentrations of POM and surface sediments from earlier studies in Figure 8a. Sediment samples showed generally higher HMW *n*‐alkane concentrations than POM, also along transects of Ob and Yenisey rivers to the Kara Sea (Fernandes & Sicre, 2000). Values for POM there were on average lower than what we observed for Kolyma and Y3, with no marked differences between samples collected in purely riverine, mixing zone, and marine environments. For POM samples from the Mackenzie river (Yunker et al., 2002) and outer delta in the Beaufort Sea (Tolosa et al., 2013) a similar decrease in HMW *n*‐alkane concentrations from river to sea was observed as found between Kolyma river and East Siberian Sea. The values for POM from smaller Canadian arctic rivers (Yunker et al., 2002) and the Kalix river in subarctic Sweden (van Dongen et al., 2008) were lower than our observations for Kolyma and Y3.

The ratio of low‐molecular‐weight (LMW, chain‐lengths of 16 and 18 carbon atoms) to HMW *n*‐alkanoic acids provides a qualitative measure for in‐stream production. Values are on average higher for Y3 (Figure 8b). For Kolyma, these ratios exhibit a negative correlation with δ^13^C (i.e., more depleted ^13^C for higher LMW/HMW *n*‐alkanoic acid ratios, *R* ^2^ = 0.55, *p* < 0.001), supporting the assumption of an aquatic POM endmember with a relatively depleted δ^13^C signature.

Both HMW *n*‐alkane and *n*‐alkanoic acid per liter concentrations correlate with POC concentrations, yet more strongly for Kolyma than for Y3 (Figures 8c and 8d). These patterns suggest that Kolyma‐POM is dominated by terrigenous sources, although the nonzero intercept hints toward a small POM contribution from aquatic production. This aquatic POM contribution is likely higher for Y3, where correlations between the biomarker and POC concentrations are weaker. A similar correlation of HMW *n*‐alkanes with organic carbon concentrations has also been observed for sediments from the Yenisey and Ob rivers (Fernandes & Sicre, 2000), as well as for HMW *n*‐alkanes with suspended particulate matter for the Mackenzie River (Yunker et al., 2002).

### 4.3 POM: Degradation Status

**Figure 9:**
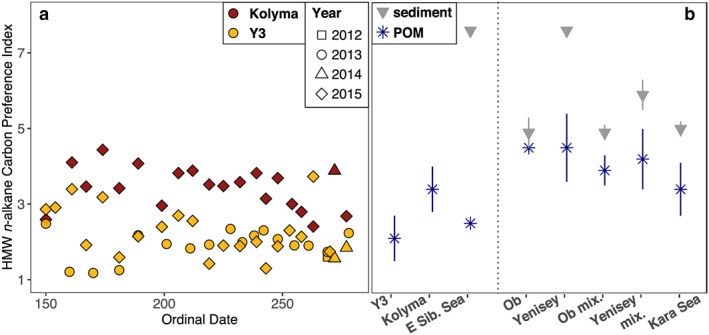
Leaf wax lipid degradation proxy HMW *n*‐alkane Carbon Preference Index for (a) Kolyma (red) and Y3 (yellow) and (b) average values for Y3 and Kolyma from this study compared to values from the Kolyma paleoriver (East Siberian Sea, Vonk et al., 2010) and Ob and Yenisey river to Kara Sea transects (Fernandes & Sicre, 2000) with blue stars depicting POM and gray triangles surface sediment samples. Vertical lines resemble ±σ. Lower Carbon Preference Index values generally indicate more degraded material.

The carbon preference index (CPI), which denotes the ratio of odd‐to‐even carbon chain homologues, can serve as an indicator for the degradation status of the wax lipids. Fresh plant material shows a strong odd‐over‐even dominance for *n*‐alkanes (the opposite is true for *n*‐alkanoic acids) (Bray & Evans, 1961). This distinct pattern fades with ongoing decomposition, and thus higher CPI values indicate presence of fresher material. For Kolyma‐POM, HMW *n*‐alkane CPI values range from 2.41 to 4.44, while for Y3, these values are between 1.18 and 3.73 (Figure 9a).

Another commonly applied degradation proxy of the plant wax lipids is the ratio of HMW *n*‐alkanoic acids to *n*‐alkanes as alkanes are generally considered more recalcitrant than their acid counterparts. A lower ratio thus implies greater decomposition. Values for Kolyma‐POM fall between 0.10 and 1.98 with no clear seasonal pattern, whereas those for Y3 are slightly lower (0.03 to 1.89, Figure S5C). While the difference between the two sites is more pronounced for the HMW *n*‐alkane CPI, both lipid degradation proxies suggest that POM for Y3 is more degraded than for Kolyma, with no marked seasonal trends evident for either site.

The *n*‐alkane CPI value measured for POM from East Siberian Sea surface waters close to the Kolyma delta (2.46, Vonk et al., 2010) is similar to the ranges observed here (Kolyma: 3.4 ± 0.6, Y3: 2.1 ± 0.6), while the CPI is much higher (7.56) for the surface sediments collected at the same location (Figure 9b). The same pattern is displayed by the HMW *n*‐alkanoic acid to *n*‐alkane ratio: 1.10 for POM, similar to the values observed here, and 3.15 in the surface sediment sample, higher than any of the Kolyma or Y3 samples (Figure S5C). This suggests preferential burial of relatively undegraded yet old OM, which has been explained by contrasting signals for riverine (relatively young, yet degraded) and coastal erosion inputs (relatively old, yet undegraded) (Vonk et al., 2010). A similar explanation may also hold for the differences observed here between river and stream systems. The older, less degraded POM for Kolyma is likely derived to a larger part from erosion of the river banks and thermokarst features along the river, while the younger, more degraded POM for Y3 may be more influenced by contemporary vegetation and top soil OM, which is more readily decomposed in the stream. Samples from the Ob and Yenisey transects to the Kara Sea also displayed generally higher CPI values for sediments than for POM (Fernandes & Sicre, 2000, Figure 9b). Riverine and mixing zone POM CPI values were on average higher than our observations for the Kolyma and Y3, while POM from the Kara Sea was similar to Kolyma‐POM (3.4 ± 0.7). The trend from fresher to more degraded wax lipids from river to marine POM thus holds for all three major arctic rivers (Ob, Yenisey, Kolyma), yet the headwater stream Y3 does not follow this pattern. This may reflect the relatively large influence of predegraded POM from the seasonally thawing active layer in this small catchment.

## 5 Summary and Conclusions

This high‐resolution time‐series, comprising a total of 36 POM samples for the lower Kolyma mainstem and 35 POM samples for headwater stream Y3 collected during the open‐water (thaw) season in 2013 and 2015, offers detailed insights into the fluvial carbon dynamics of a major, permafrost‐dominated arctic watershed. Auxiliary measurements show marked differences between the two sampling sites of this study: the smaller Y3 is more strongly influenced by regional precipitation and DOC leaching from the underlying soils while the Kolyma river carries a higher POC load, likely stemming from active river bank erosion, especially during the freshet. Carbon isotope data imply a mixture of recent vegetation, active layer soil OM, possibly some Holocene permafrost soil OM, and in‐stream aquatic productivity as POM sources for Y3, whereas for the Kolyma, Holocene and Pleistocene permafrost soil contributions appear to be larger, with relatively less in‐stream production. However, no clear input from Yedoma permafrost was visible and ^14^C signatures did not display any seasonal trends that could be related to soil thaw depth. While Kolyma‐POM is generally older than Y3‐POM, biomarker degradation proxies suggest plant wax‐derived components are less decomposed than in the younger POM of Y3. Similar contrasts were found between POM and surface sediments collected outside the Kolyma delta (Vonk et al., 2010). Biomarker concentrations did not show clear seasonal patterns but were on average higher for Kolyma than Y3 and comparable to those of POM from the East Siberian Sea (Vonk et al., 2010). While this study improves our knowledge of the composition of present‐day POM delivery, the degradability of permafrost‐derived POM as well as the environmental, physical, and chemical factors influencing decomposition remain open questions. More research is needed to fully understand its role in ongoing Arctic change.

## Supporting information

Supporting Information S1
