# Supplementary material for: Particulate Organic Matter Dynamics in a Permafrost Headwater Stream and the Kolyma River Mainstem
Source: J Geophys Res Biogeosci. 2020 Feb 21;125(2):e2019JG005511. doi: 10.1029/2019JG005511 (PMC7375038; doi:10.1029/2019JG005511)
Supplement: Supplementary file 1 — Supporting Information S1 [file JGRG-125-e2019JG005511-s001.pdf]

Journal of Geophysical Research - Biogeosciences

Supporting Information for

Particulate organic matter dynamics in a permafrost headwater stream and the Kolyma River mainstem

Lisa Bröder[1,2], Anya Davydova[3], Sergey Davydov[3], Nikita Zimov[3], Negar Haghipour[2,4], Timothy I. Eglinton[2], and Jorien E. Vonk[1]

[1]Department of Earth Sciences, Vrije Universiteit Amsterdam, Amsterdam, The Netherlands.

[2]Geological Institute, Swiss Federal Institute of Technology (ETH), Zürich, Switzerland.

[3]Northeast Science Station, Pacific Geographical Institute, Far East Branch, Russian Academy of Sciences, Cherskiy, Republic of Sakha, Russia.

[4]Laboratory of Ion Beam Physics, Swiss Federal Institute of Technology (ETH), Zürich, Switzerland.

Contents of this file

Figures S1 to S5
Tables S1 to S8

Introduction

This file contains five figures and eight tables:
- **Figure S1** visualizes meteorological data (daily average temperature and precipitation) measured at Cherskiy airport together with water properties pH and specific conductivity for Kolyma river and Y3 stream at the time of sampling.
- **Figure S2** shows hydrogen water isotope (δ^2H) and deuterium excess (d) values for Kolyma and Y3 water samples.
- **Figure S3** displays the relationship between the Kolyma river discharge at the sampling day and the concentrations of particulate and dissolved organic carbon concentrations (POC and DOC, respectively) for the collected water samples.
- **Figure S4** shows Total Particulate Nitrogen (TPN) concentrations and molar carbon-to-nitrogen ratios (POC/TPN) for Kolyma and Y3.
- **Figure S5** displays ratios of high-molecular weight (HMW) ***n***-alkanoic acid and ***n***-alkane concentrations, a lipid biomarker degradation proxy, for Kolyma and Y3.
- **Tables S1 and S2** contain water properties (T, pH, EC, δ^2H, $\delta^{18}O$, d) for Kolyma and Y3, respectively.

- **Tables S3 and S4** list DOC, total dissolved nitrogen (TDN), POC and TPN for Kolyma and Y3 water samples, respectively.
- **Tables S5 and S6** provide stable and radiocarbon isotopic data for POC of Kolyma and Y3 water samples, respectively.
- **Tables S7 and S8** summarize POC lipid biomarker results for Kolyma and Y3 water samples, respectively.

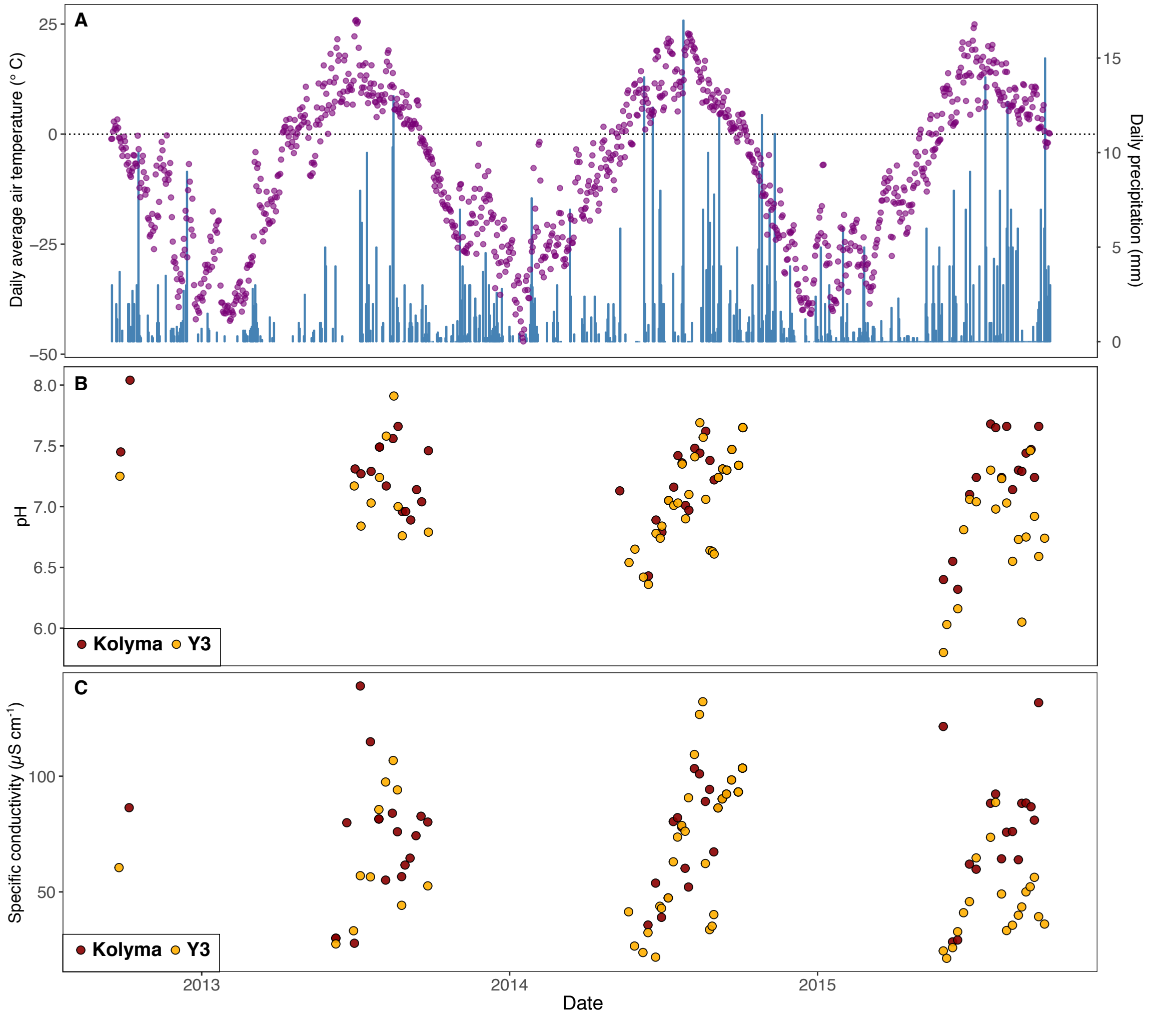

Figure S1. (**A**) Daily average air temperatures (purple filled circles) and precipitation (blue bars) recorded at Cherskiy airport (Cherskiy Weather Station, unpublished data 1980 - 2018). (**B**) Kolyma river (red) and Y3 stream (yellow) water pH and (**C**) specific conductivity measured when sampling.

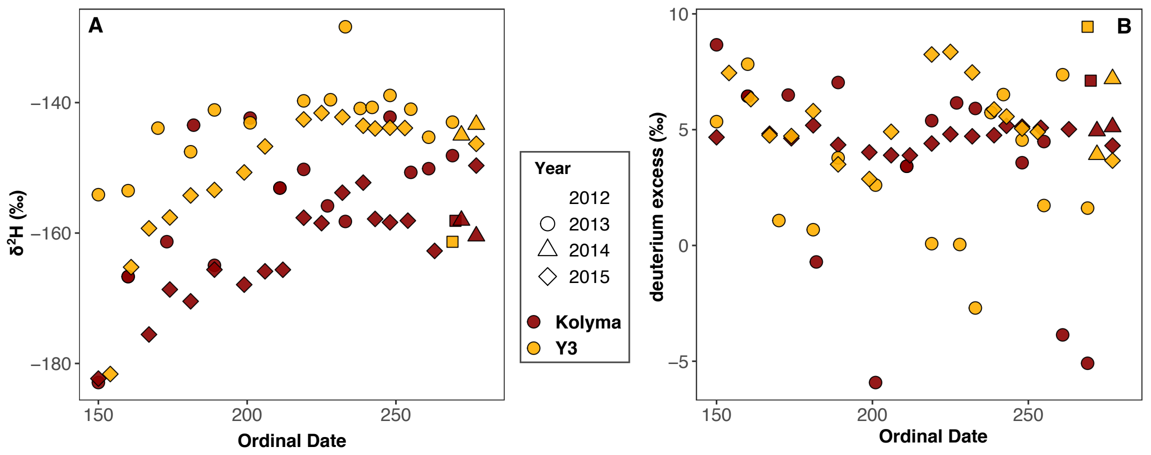

Figure S2. (**A**) Hydrogen water isotope values δ^2H for Kolyma (red) and Y3 (yellow) increase from very low values during the freshet and stabilize around mid-August. Y3 waters have on average higher (less depleted) values than Kolyma waters. (**B**) Deuterium excess d (calculated as $d = \delta^2H - 8 * \delta^{18}O$) for Kolyma (red) and Y3 (yellow) are similar and do not show clear seasonal trends.

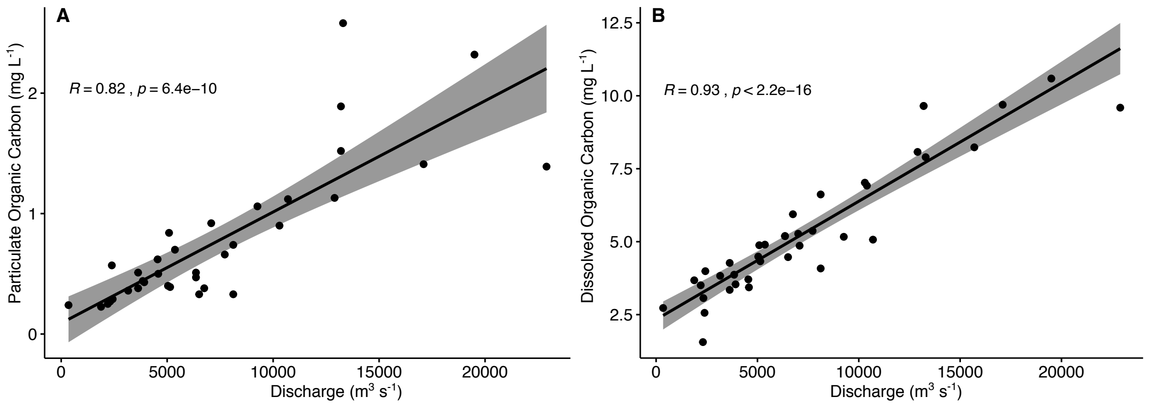

Figure S3. (**A**) Particulate Organic Carbon (POC) and (**B**) Dissolved Organic Carbon (DOC) concentrations of Kolyma water samples are strongly correlated with the daily average discharge of the river for the sampling date.

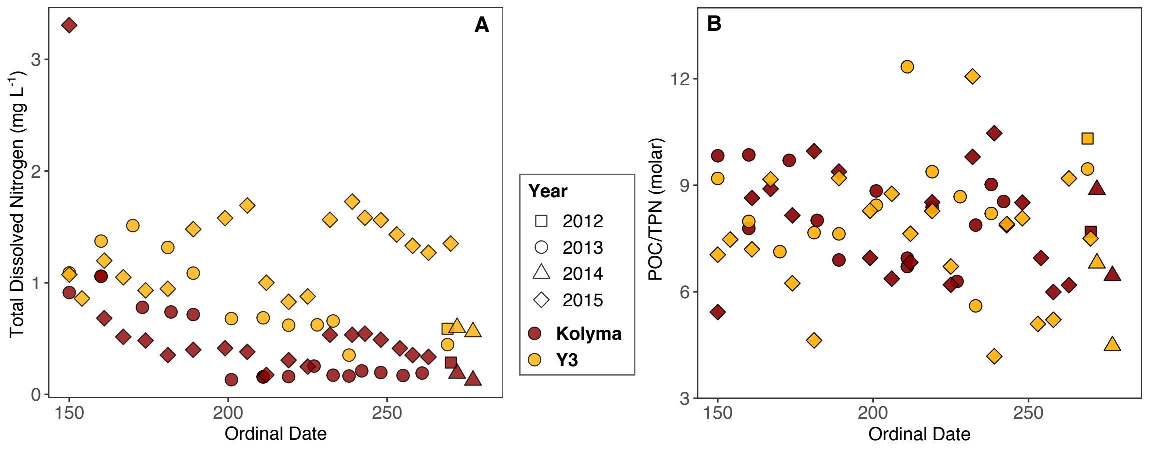

Figure S4. (**A**) Total Particulate Nitrogen concentrations for Kolyma (red) show a decreasing trend from higher values during the spring flood, while for Y3 (yellow) there is no clear trend but concentrations are on average higher than for Kolyma. (**B**) The molar carbon-to-nitrogen ratio for POM (POC/TPN) does not display seasonal trends or differences between the sites.

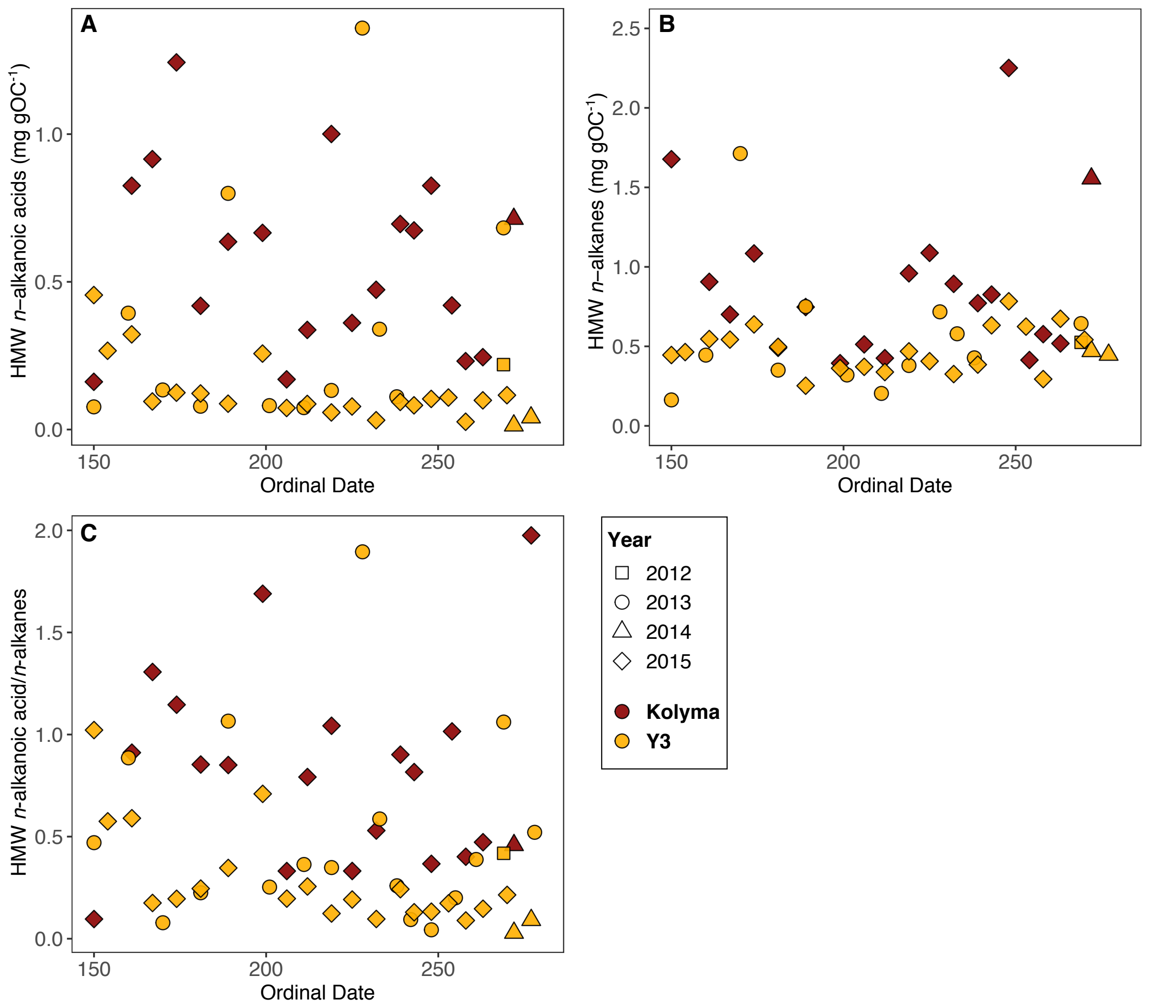

Figure S5. Concentrations of (**A**) high-molecular-weight (HMW) n-alkanes and (**B**) n-alkanoic acids for Kolyma (red) and Y3 (yellow). (**C**) The ratio of high-molecular weight (HMW) *n*-alkanoic acid and *n*-alkane concentrations for Kolyma (red) and Y3 (yellow) shows no clear seasonal trend but is on average higher for Kolyma, suggesting relatively more degraded material.

Date	T (˚C)	pH	EC (µS cm^{-1})	$\delta^{18}O$ (‰)	δ^2H (‰)	d (‰)
2012-09-26	2.7	7.45		-20.7	-158.1	7.11
2012-10-07	2	8.04	86.4	-20.8	-158.8	7.66
2013-05-30				-23.9	-182.9	8.66
2013-06-09	10.1		30	-21.6	-166.7	6.44
2013-06-09	10.1		30	-21.6	-166.7	6.44
2013-06-22	13		79.9	-21.0	-161.3	6.50
2013-07-01	15	7.31	27.8	-17.8	-143.5	-0.71
2013-07-08	14.7	7.27	139	-21.5	-165.0	7.04
2013-07-20	14.9	7.29	114.9	-17.1	-142.4	-5.92
2013-07-30	13.2	7.49	81.5	-19.6	-153.1	3.42
2013-07-30	13.2	7.49	81.5	-19.6	-153.1	3.42
2013-08-07	11.9	7.17	55.1	-19.5	-150.2	5.39
2013-08-15	12.9	7.56	84	-20.2	-155.8	6.16
2013-08-21	13.5	7.66	76	-20.5	-158.2	5.92
2013-08-26	10.2	6.96	56.6			
2013-08-30	8.1	6.96	61.6			
2013-09-05	7.10	6.890	64.6	-18.2	-142.2	3.57
2013-09-12	5.70	7.140	74.3	-19.4	-150.7	4.49
2013-09-18	5.60	7.040	82.7	-18.3	-150.1	-3.86
2013-09-26	3.60	7.460	80.2	-17.9	-148.1	-5.09
2013-11-13				-20.7	-159.2	6.22
2014-09-29	3.6	7.34	93.2	-20.38	-158.06	4.94
2014-10-04	3.5	7.65	103.5	-20.70	-160.47	5.12
2014-10-11				-20.78	-161.57	4.64
2015-05-30	0.2	6.4	121.5	-23.37	-182.32	4.67
2015-06-10	3.8	6.55	28.4			
2015-06-16	9.2	6.32	29.2	-22.55	-175.56	4.82
2015-06-23				-21.66	-168.66	4.63
2015-06-30	14.8	7.1	62	-21.96	-170.47	5.19
2015-07-08	16.3	7.24	59.8	-21.24	-165.61	4.34
2015-07-18				-21.49	-167.91	4.02
2015-07-25	16.6	7.68	88.3	-21.22	-165.87	3.89
2015-07-31	16.3	7.65	92.3	-21.19	-165.63	3.89
2015-08-07	13.4	7.24	64.3	-20.26	-157.65	4.40
2015-08-13	13	7.66	75.8	-20.41	-158.49	4.80
2015-08-20	10.7	7.14	76.1	-19.82	-153.81	4.71
2015-08-27	8.3	7.3	63.9	-19.63	-152.27	4.76
2015-08-31	9.5	7.29	88.3	-20.38	-157.84	5.17
2015-09-05	8.7	7.44	88.4	-20.44	-158.36	5.13

2015-09-11	8.6	7.47	86.8	-20.40	-158.10	5.08
2015-09-15	8.7	7.24	81			
2015-09-20	7.3	7.66	131.8	-20.97	-162.74	5.02
2015-10-04				-19.2	-149.7	4.31

Table S1. Kolyma River water properties at time of sampling: water temperature T, specific conductivity EC, water isotopes ^{18}O and ^{2}H and deduced deuterium excess d.

Date	T (˚C)	pH	EC (µS cm^{-1})	δ^{18}O (‰)	δ^{2}H (‰)	d (‰)
2012-09-25	1.6	7.25	60.5	-21.35	-161.3	9.44
2013-05-30				-19.93	-154.1	5.35
2013-06-09	8.8		27.5	-20.17	-153.5	7.83
2013-06-19				-18.12	-143.9	1.08
2013-06-30	12.7	7.17	33.2	-18.53	-147.5	0.68
2013-07-08	17.1	6.84	57	-18.11	-141.1	3.78
2013-07-20	12.2	7.03	56.5	-18.21	-143.1	2.61
2013-07-30	11	7.24	85.6			
2013-08-07	11.2	7.58	97.5	-17.48	-139.7	0.08
2013-08-16	10	7.91	106.8	-17.45	-139.6	0.04
2013-08-21	10.5	7.00	94.10	-15.71	-128.4	-2.70
2013-08-26	7.1	6.76	44.2	-18.33	-140.9	5.74
2013-08-30				-18.41	-140.7	6.52
2013-09-05				-17.93	-138.9	4.55
2013-09-12				-17.84	-141.0	1.72
2013-09-18				-19.09	-145.3	7.37
2013-09-26	0.3	6.79	52.6	-18.08	-143.0	1.61
2014-09-29	3.6	7.34	93.2	-18.62	-145.0	3.92
2014-10-04	3.5	7.65	103.5	-18.82	-143.4	7.20
2015-05-30	0.1	5.8	24.5			
2015-06-03	4.7	6.03	21.3	-23.63	-181.6	7.45
2015-06-10	2.3		25.9	-21.44	-165.2	6.32
2015-06-16	8.4	6.16	32.8	-20.50	-159.3	4.75
2015-06-23	10.6	6.81	41	-20.29	-157.6	4.72
2015-06-30	11.8	7.06	45.8	-20.00	-154.2	5.80
2015-07-08	12.5	7.04	64.7	-19.61	-153.4	3.50
2015-07-18				-19.20	-150.7	2.87
2015-07-25	16	7.3	73.6	-18.95	-146.7	4.91
2015-07-31	13.5	6.98	88.7			
2015-08-07	8.9	7.23	49.1	-18.85	-142.6	8.25
2015-08-13	7.4	7.03	33.3	-18.74	-141.6	8.36

2015-08-20	6.6	6.55	35.6	-18.71	-142.2	7.47
2015-08-27	4	6.73	39.9	-18.68	-143.5	5.87
2015-08-31	6.8	6.05	43.5	-18.69	-144.0	5.57
2015-09-05	4.6	6.75	50	-18.61	-143.8	5.05
2015-09-10	2.1	7.46	52.2	-18.60	-143.9	4.90
2015-09-15	4.5	6.92	56.3			
2015-09-20	1.8	6.59	39.3			
2015-09-27	0.1	6.74	36.1			
2015-10-04				-18.75	-146.3	3.66

Table S2. Y3 stream water properties at time of sampling: water temperature T, specific conductivity EC, water isotopes ^{18}O and ^{2}H and deduced deuterium excess d.

Date	DOC (mg L^{-1})	TDN (mg L^{-1})	POC (mg L^{-1})	TPN (mg L^{-1})
2012-09-26	3.83	0.29	0.36	0.055
2012-10-07	3.51	0.15	0.25	0.047
2013-05-30	9.59	0.91	1.39	0.165
2013-06-09	9.65	1.06	1.89	0.283
2013-06-09	9.65	1.06	1.52	0.180
2013-06-22	7.02	0.78	0.9	0.108
2013-07-01	5.17	0.74	1.06	0.154
2013-07-08	4.47	0.72	0.33	0.056
2013-07-20	3.71	0.13	0.62	0.082
2013-07-30	5.19	0.16	0.47	0.079
2013-07-30	5.19	0.16	0.51	0.089
2013-08-07	5.94	0.16	0.38	0.053
2013-08-15	5.37	0.25	0.66	0.122
2013-08-21	4.08	0.17	0.33	0.049
2013-08-26	6.62	0.16	0.74	0.096
2013-08-30	8.08	0.21	1.13	0.154
2013-09-05	8.23	0.20		
2013-09-12	6.92	0.17		
2013-09-18	5.27	0.19		
2013-09-26				
2013-11-13				
2014-09-29	2.56	0.19	0.57	0.075
2014-10-04	1.56	0.13	0.27	0.049
2015-05-30	2.73	3.31	0.24	0.052
2015-06-10	10.59	0.68	2.32	0.313
2015-06-16	9.69	0.52	1.41	0.185

2015-06-23	7.90	0.48	2.58	0.369
2015-06-30	5.07	0.35	1.12	0.131
2015-07-08	4.86	0.40	0.92	0.114
2015-07-18	3.87	0.41	0.44	0.074
2015-07-25	3.54	0.38	0.43	0.079
2015-07-31	3.35	0.17	0.38	0.065
2015-08-07	3.43	0.31	0.5	0.068
2015-08-13	4.33	0.25	0.39	0.073
2015-08-20	4.88	0.53	0.84	0.100
2015-08-27	4.90	0.53	0.7	0.078
2015-08-31	4.49	0.54	0.4	0.059
2015-09-05	4.27	0.49	0.51	0.070
2015-09-11	3.99	0.41	0.29	0.049
2015-09-15	3.68	0.35	0.23	0.044
2015-09-20	3.07	0.34	0.28	0.053

Table S3. Concentrations of dissolved organic carbon (DOC), total dissolved nitrogen (TDN), particulate organic carbon (POC) and total particulate nitrogen (TPN) for Kolyma water samples.

Date	DOC (mg L^{-1})	TDN (mg L^{-1})	POC (mg L^{-1})	TPN (mg L^{-1})
2012-09-25	12.81	0.59	0.26	0.029
2013-05-30	15.31	1.09	0.74	0.094
2013-06-09	16.52	1.37	0.20	0.029
2013-06-19	21.49	1.51	0.13	0.021
2013-06-30	19.41	1.32	0.17	0.026
2013-07-08	17.68	1.09	0.33	0.050
2013-07-20	20.69	0.68	0.25	0.035
2013-07-30	20.26	0.69	0.38	0.036
2013-08-07	22.16	0.62	0.39	0.049
2013-08-16	21.57	0.62	0.30	0.040
2013-08-21	19.92	0.66	0.28	0.059
2013-08-26	17.28	0.35	0.19	0.027
2013-09-26	15.74	0.45	0.16	0.020
2014-09-29	17.69	0.60	0.14	0.024
2014-10-04	18.12	0.56	0.14	0.038
2015-05-30	20.96	1.07	0.87	0.144
2015-06-03	14.97	0.86	0.77	0.120
2015-06-10	20.81	1.20	0.23	0.038
2015-06-16	17.87	1.05	0.20	0.026
2015-06-23	17.48	0.93	0.14	0.025

2015-06-30	18.45	0.95	0.17	0.044
2015-07-08	18.70	1.48	0.31	0.039
2015-07-18	17.10	1.58	0.26	0.037
2015-07-25	17.18	1.69	0.32	0.043
2015-07-31	18.90	1.00	0.41	0.063
2015-08-07	17.12	0.83	0.26	0.036
2015-08-13	19.67	0.88	0.33	0.057
2015-08-20	20.28	1.56	0.35	0.034
2015-08-27	18.68	1.73	0.20	0.056
2015-08-31	18.77	1.58	0.15	0.023
2015-09-05	18.55	1.56	0.14	0.020
2015-09-10	17.16	1.43	0.13	0.029
2015-09-15	16.21	1.33	0.15	0.033
2015-09-20	20.69	1.27	0.31	0.039
2015-09-27	21.52	1.35	0.15	0.023

Table S4. Concentrations of dissolved organic carbon (DOC), total dissolved nitrogen (TDN), particulate organic carbon (POC) and total particulate nitrogen (TPN) with carbon isotopic values for POC of Y3 water samples.

Date	$\delta^{13}C$ (‰)	$\Delta^{14}C$ (‰)	ETH lab nr.	Fm	±
2012-09-26	-28.60	-261.4	87003.1.1	0.7297	0.0074
2012-10-07	-30.48	-213.5	87002.1.1	0.7731	0.0077
2013-05-30	-31.77	-109.2	86997.1.1	0.8667	0.0079
2013-06-09	-28.95	-233.8	86996.1.1	0.8488	0.0079
2013-06-09	-28.73	-129.9	86998.1.1	0.7546	0.0071
2013-06-22	-28.31	-343.7	86991.1.1	0.6559	0.0067
2013-07-01	-32.60	-212.4	87000.1.1	0.7741	0.0074
2013-07-08	-29.06	-301.3	86984.1.1	0.6915	0.0071
2013-07-20	-28.88	-306.9	87008.1.1	0.6890	0.0066
2013-07-30	-27.38	-385.7	86989.1.1	0.6340	0.0065
2013-07-30	-27.43	-367.2	86988.1.1	0.6172	0.0063
2013-08-07	-27.45	-436.9	86985.1.1	0.5719	0.0061
2013-08-15	-27.88	-392.5	87001.1.1	0.6119	0.0062
2013-08-21	-27.82	-359.6	87004.1.1	0.6414	0.0069
2013-08-26	-27.34	-416.9	86975.1.1	0.5898	0.0000
2013-08-30	-26.96				
2013-09-05		-302.0	86986.1.1	0.6933	0.0074
2013-09-12		-355.4	86553.1.1	0.6372	0.0064
2013-09-18		-366.8	86555.1.1	0.7879	0.0077
2013-09-26		-182.3	86554.1.1	0.6304	0.0065

2013-11-13		-166.2	86556.1.1	0.8035	0.0080
2014-09-29	-28.68	-404.1	86993.1.1	0.6015	0.0062
2014-10-04	-30.88	-246.8	87009.1.1	0.7351	0.0077
2015-05-30		-183.2	86999.1.1	0.7836	0.0073
2015-06-10	-26.71	-297.6	87007.1.1	0.6977	0.0068
2015-06-16	-28.57	-224.6	86980.1.1	0.7637	0.0078
2015-06-23	-27.95	-438.0	86981.1.1	0.5709	0.0067
2015-06-30	-27.55	-378.7	86992.1.1	0.6245	0.0063
2015-07-08	-28.91	-353.0	86987.1.1	0.6476	0.0067
2015-07-18	-31.43	-212.3	86983.1.1	0.7741	0.0079
2015-07-25	-31.55	-213.2	86977.1.1	0.7696	0.0094
2015-07-31	-31.75	-199.3	87005.1.1	0.7846	0.0078
2015-08-07	-29.04	-390.8	86990.1.1	0.6135	0.0064
2015-08-13	-30.16	-288.1	86982.1.1	0.7026	0.0070
2015-08-20	-27.43	-431.2	86995.1.1	0.5771	0.0061
2015-08-27	-27.41	-375.6	86994.1.1	0.6272	0.0062
2015-08-31	-27.80				
2015-09-05	-28.58				
2015-09-11	-30.99	-205.4	86974.1.1	0.7714	0.0072
2015-09-15		-193.3	87010.1.1	0.7791	0.0071
2015-09-20	-28.77	-321.6	87006.1.1	0.6753	0.0065

Table S5. Stable and radiocarbon isotopic values ($\delta^{13}C$ and $\Delta^{14}C$, respectively) for POC of Kolyma water samples. For radiocarbon measurements, reference numbers of the ETH laboratories are given, as well as the raw data for fraction modern (Fm) and its standard deviation (±).

Date	$\delta^{13}C$ (‰)	$\Delta^{14}C$ (‰)	ETH lab nr.	Fm	±
2012-09-25	-29.34	-34.8	86535.1.1	0.9247	0.0085
2013-05-30	-29.60	-25.4	86523.1.1	0.9349	0.0090
2013-06-09	-30.08	-29.1	86551.1.1	0.9298	0.0088
2013-06-19	-28.88	-33.2	86537.1.1	0.9095	0.0084
2013-06-30	-31.04				
2013-07-08	-31.05	-42.3	86519.1.1	0.8995	0.0082
2013-07-20		-2.7	86520.1.1	0.9067	0.0084
2013-07-30	-35.14	-173.3	86518.1.1	0.7915	0.0077
2013-08-07	-30.24	-115.9	86533.1.1	0.8525	0.0081
2013-08-16	-31.19	-47.7	86547.1.1	0.9107	0.0084
2013-08-21	-29.33	-16.4	86552.1.1	0.9322	0.0086
2013-08-26	-28.29	-260.9	86540.1.1	0.7204	0.0074
2013-09-26	-29.49	-14.3	86522.1.1	0.9290	0.0086
2014-09-29		-94.6	86539.1.1	0.8375	0.0080

2014-10-04		-60.9	86536.1.1	0.8461	0.0081
2015-05-30	-26.34	61.6	86544.1.1	1.0126	0.0089
2015-06-03	-26.76	-25.6	86534.1.1	0.9337	0.0088
2015-06-10		-130.9	86549.1.1	0.8203	0.0080
2015-06-16		-85.2	86550.1.1	0.8313	0.0078
2015-06-23		-72.7	86532.1.1	0.8314	0.0076
2015-06-30		-207.7	86530.1.1	0.7467	0.0072
2015-07-08	-29.71	-56.4	86542.1.1	0.8962	0.0084
2015-07-18	-29.32	-87.7	86531.1.1	0.8557	0.0081
2015-07-25	-29.93	-33.8	86541.1.1	0.9165	0.0083
2015-07-31	-30.96	-54.9	86538.1.1	0.9032	0.0084
2015-08-07		-49.5	86545.1.1	0.9039	0.0084
2015-08-13	-28.45	-63.1	86521.1.1	0.8811	0.0086
2015-08-20	-28.94	-75.5	86543.1.1	0.8696	0.0083
2015-08-27		-51.8	86529.1.1	0.8836	0.0080
2015-08-31		-82.4	86525.1.1	0.8276	0.0080
2015-09-05		-151.5	86526.1.1	0.7822	0.0075
2015-09-10		-84.0	86528.1.1	0.8294	0.0077
2015-09-15		-23.8	86524.1.1	0.8664	0.0082
2015-09-20	-27.51	-202.4	86527.1.1	0.7702	0.0075
2015-09-27		-127.4	86548.1.1	0.8023	0.0082

Table S6. Stable and radiocarbon isotopic values ($\delta^{13}C$ and $\Delta^{14}C$, respectively) for POC of Y3 water samples. For radiocarbon measurements, reference numbers of the ETH laboratories are given, as well as the raw data for fraction modern (Fm) and its standard deviation (±).

**Date**	**HMW *n*-alk (µg gOC^{-1})**	**HMW *n*-alk acid (µg gOC^{-1})**	**LMW/HMW acids**	**CPI alk**
2014-09-29	1556	713	2.33	3.88
2015-05-30	1677	162	32.57	2.59
2015-06-10	906	825	3.13	4.10
2015-06-16	701	915	5.72	3.46
2015-06-23	1085	1243	1.93	4.44
2015-06-30	491	419	1.31	3.42
2015-07-08	747	636	1.87	4.07
2015-07-18	394	666	11.01	2.96
2015-07-25	512	170	16.50	3.82
2015-07-31	426	337	15.33	3.88
2015-08-07	959	1000	2.16	3.52
2015-08-13	1088	361	7.38	3.48
2015-08-20	893	473	2.37	3.58

2015-08-27	772	696	2.85	3.82
2015-08-31	826	674	3.71	3.14
2015-09-05	2251	826	3.53	3.69
2015-09-11	414	420	7.15	3.00
2015-09-15	577	232	15.82	2.79
2015-09-20	518	245	15.67	2.41
2015-10-04			5.63	2.68

Table S7. Kolyma River POC biomarker results: Concentrations of high-molecular weight ***n***-alkanes (HMW *n*-alk) and high-molecular weight *n*-alkanoic acids (HMW *n*-alk acid) normalized to organic carbon concentrations, ratios of low- to high-molecular weight *n*-alkanoic acids (LMW/HMW acids, i.e., concentrations of homologues with carbon chain lengths of 16 and 18 divided by those with 24, 26, 28 and 30 carbon atoms) and carbon preference indices (CPI alk, i.e., HMW *n*-alkanes with odd chain lengths 23-31 divided by the average of the sum of even chain lengths 22-30 and 24-32).

Date	HMW *n*-alk (µg gOC^{-1})	HMW *n*-alk acid (µg gOC^{-1})	LMW/HMW acids	CPI alk
2012-09-25	525	220	13.48	1.60
2013-05-30	163	77	22.28	2.48
2013-06-09	445	394	33.66	1.21
2013-06-19	1713	134	42.43	1.18
2013-06-30	350	79	68.62	1.25
2013-07-08	750	800	25.85	2.17
2013-07-20	320	81	36.19	1.94
2013-07-30	204	74	31.44	1.83
2013-08-07	380	132	24.64	1.93
2013-08-16	717	1359	4.48	2.34
2013-08-21	579	340	28.47	1.99
2013-08-26	428	111	76.04	2.17
2013-08-30			33.77	2.30
2013-09-05			81.98	2.07
2013-09-12			57.92	1.91
2013-09-18			45.82	1.90
2013-09-26	643	683	35.46	1.73
2013-10-05			28.75	2.23
2014-09-29	470	13	63.65	1.57
2014-10-04	447	41	42.21	1.85
2015-05-30	445	455	6.98	2.86
2015-06-03	464	267	11.22	2.91
2015-06-10	546	322	6.03	3.39
2015-06-16	543	95	18.79	1.92

2015-06-23	638	125	19.71	3.18
2015-06-30	497	122	31.08	1.59
2015-07-08	252	87	29.82	2.15
2015-07-18	362	257	10.29	2.40
2015-07-25	371	73	27.15	2.69
2015-07-31	338	87	23.58	2.55
2015-08-07	469	58	19.79	1.42
2015-08-13	407	78	28.91	1.90
2015-08-20	326	31	32.62	1.89
2015-08-27	385	93	21.36	2.00
2015-08-31	632	82	26.81	1.30
2015-09-05	783	104	23.13	1.88
2015-09-10	624	108	35.13	2.30
2015-09-15	294	26	78.70	2.14
2015-09-20	674	99	11.24	3.73
2015-09-27	542	116	20.02	1.74

Table S8. Y3 stream POC biomarker results: Concentrations of high-molecular weight ***n***-alkanes (HMW *n*-alk) and high-molecular weight *n*-alkanoic acids (HMW *n*-alk acid) normalized to organic carbon concentrations, ratios of low- to high-molecular weight *n*-alkanoic acids (LMW/HMW acids, i.e., concentrations of homologues with carbon chain lengths of 16 and 18 divided by those with 24, 26, 28 and 30 carbon atoms) and carbon preference indices (CPI alk, i.e., HMW *n*-alkanes with odd chain lengths 23-31 divided by the average of the sum of even chain lengths 22-30 and 24-32).
